# Low-Intensity Guided Help Through Mindfulness (LIGHTMIND): study protocol for a randomised controlled trial comparing supported mindfulness-based cognitive therapy self-help to supported cognitive behavioural therapy self-help for adults experiencing depression

**DOI:** 10.1186/s13063-020-04322-1

**Published:** 2020-05-04

**Authors:** Clara Strauss, Amy Arbon, Michael Barkham, Sarah Byford, Rebecca Crane, Richard de Visser, Margaret Heslin, Anna-Marie Jones, Fergal Jones, Laura Lea, Glenys Parry, Claire Rosten, Kate Cavanagh

**Affiliations:** 1 School of Psychology, University of Sussex, Pevensey Building, Falmer, BN1 9QH UK; 2 Sussex Partnership NHS Foundation Trust, R&D Department, Sussex Education Centre, Nevill Avenue, Hove, BN3 7HZ UK; 3 Brighton and Sussex University Hospitals NHS Trust, Royal Sussex County Hospital, Eastern Road, Brighton, BN2 5BE UK; 4 Brighton & Sussex Clinical Trials Unit, Bevendean House, University of Brighton, Falmer, BN1 9PH UK; 5 Clinical Psychology Unit, Department of Psychology, University of Sheffield, S10 2TP, Sheffield, UK; 6 King’s Health Economics Research Group and Health Service and Population Research Department, Institute of Psychiatry, Psychology & Neuroscience, King’s College London, London, SE5 8AF UK; 7 Centre for Mindfulness Research and Practice, School of Psychology, Bangor University, Bangor, Gwynedd LL57 2AS UK; 8 School of Health Sciences, University of Brighton, Village Way, Brighton, BN1 9PH UK; 9 Canterbury Christ Church University, Salmons Institute for Applied Psychology, Lucy Fildes Building, 1 Meadow Road, Tunbridge Wells, TN1 2YG UK; 10 School of Health and Related Research, University of Sheffield, S10 2TP, Sheffield, UK

**Keywords:** Depression, Mindfulness, Cognitive behavioural therapy, CBT, Mindfulness-based cognitive therapy, MBCT, Self-help, Randomised controlled trial, RCT

## Abstract

**Background:**

Depression has serious personal, family and economic consequences. It is estimated that it will cost £12.15 billion to the economy each year in England by 2026. Improving access to psychological therapies (IAPT) is the National Health Service talking therapies service in England for adults experiencing anxiety or depression. Over 1 million people are referred to IAPT every year, over half experiencing depression. Where symptoms of depression are mild to moderate, people are typically offered cognitive behavioural therapy (CBT) self-help (CBT-SH) supported by a psychological well-being practitioner. The problem is that over half of people who complete treatment for depression in IAPT remain depressed despite receiving National Institute of Health and Care Excellent recommended treatment. Furthermore, less than half of IAPT service users complete treatment. This study seeks to investigate the effectiveness of an alternative to CBT-SH. Mindfulness-based cognitive therapy (MBCT) differs from CBT in focus, approach and practice, and may be more effective with a higher number of treatment completions.

**Methods/design:**

This is a definitive randomised controlled trial comparing supported MBCT self-help (MBCT-SH) with CBT-SH for adults experiencing mild to moderate depression being treated in IAPT services. We will recruit 410 participants experiencing mild to moderate depression from IAPT services and randomise these to receive either an MBCT-based self-help workbook or a CBT-based self-help workbook. Participants will be asked to complete their workbook within 16 weeks, with six support sessions with a psychological well-being practitioner. The primary outcome is depression symptom severity on treatment completion. Secondary outcomes are treatment completion rates and measures of generalized anxiety, well-being, functioning and mindfulness. An exploratory non-inferiority analysis will be conducted in the event the primary hypothesis is not supported. A semi-structured interview with participants will guide understanding of change processes.

**Discussion:**

If the findings from this randomised controlled trial demonstrate that MBCT-SH is more effective than CBT-SH for adults experiencing depression, this will provide evidence for policy makers and lead to changes to clinical practice in IAPT services, leading to greater choice of self-help treatment options and better outcomes for service users. If the exploratory non-inferiority analysis is conducted and this indicates non-inferiority of MBCT-SH in comparison to CBT-SH this will also be of interest to policy makers when seeking to increase service user choice of self-help treatment options for depression.

**Trial registration:**

Current Controlled Trial registration number: ISRCTN 13495752. Registered on 31 August 2017 (www.isrctn.com/ISRCTN13495752).

## Background

Around 15% of adults in England experience clinically significant depression or anxiety in any week [1]. Improving access to psychological therapies (IAPT) is an initiative launched by the National Health Service (NHS) in England in 2006 that aims to improve access to psychological therapies for people experiencing anxiety and depression, with over 1.5 million people now referred to IAPT each year [2].

Depression is typically recurrent; following one episode of major depression, 50% will relapse, and after two episodes 80% relapse [3]. In addition to the impact on individuals and their families, depression is estimated to cost the economy in England £12.15 billion a year by 2026 [4]. In order to meet the needs of people experiencing depression, IAPT typically offers stepped-care [5] consisting of supported self-help at ‘step 2’ followed by, where needed, face-to-face therapy at ‘step 3’. In line with National Institute of Health and Care Excellence (NICE) guidelines [6], at step 2 people are provided with cognitive behavioural therapy (CBT) self-help (CBT-SH) materials supported by a trained psychological well-being practitioner (PWP).

However, IAPT has shown modest treatment outcomes for CBT-SH; only 41% of people receiving book-based PWP-supported CBT-SH for depression met criteria for remission in the 2018–2019 financial year [2]. In addition, this figure is for initial remission and does not consider sustained recovery [7]. Partial remission from depression is associated with a greater risk of relapse [8]. A related problem in IAPT is high rates of treatment drop-out; only 36% of people referred to IAPT in 2018–2019 completed a course of treatment [2]. Completing treatment is important because it is associated with better outcomes [9]. Moreover, costs for treatment non-completers surpass that of treatment completers in IAPT [10]. However, there is poor understanding of the reasons for non-completion [11], and this evidence gap needs addressing. Improving remission rates for depression and increasing treatment completion require urgent attention.

Mindfulness is the capacity to pay attention, intentionally and non-judgmentally, to current experience. Mindfulness-based interventions (MBIs) teach the application of mindfulness in everyday life and work by reducing rumination and worry [12]. They address well-established mechanisms which trigger and maintain depression [13]. MBIs differ from CBT in important ways: 1) CBT includes evaluating the accuracy of difficult thoughts, whereas MBIs encourage a self-compassionate, non-judgmental and accepting attitude towards experience, including unpleasant thoughts; 2) regular meditation practice (verbally guided attention towards present moment experiences) is integral to MBIs but is not included in CBT (meditation is the training ground that enables participants to experience thoughts in the moment as transient mental events [14]); and 3) participant experience is different in terms of content and goals.

Mindfulness-based cognitive therapy (MBCT) is a group therapy recommended for relapse prevention for depression in the UK by NICE [15] that includes elements of CBT for depression. Meta-analyses show that MBCT reduces the relative risk of relapse for people with a history of multiple episodes of depression by 31% [16]. Mindfulness-based group interventions, in comparison to control conditions, lead to significant reductions in depression severity for people currently depressed [17]. Thus, MBCT groups are a good candidate for not only attaining initial symptom remission but also for achieving sustained recovery and preventing relapse. However, this evidence applies to formal face-to-face MBCT groups, delivered by highly trained MBCT therapists, and we cannot assume this potential will generalise to a supported self-help intervention in a service such as IAPT.

MBCT self-help (MBCT-SH) delivered at step 2 in IAPT services has the potential to reduce the cost of delivery and to widen access to people unable or unwilling to attend a group [18]. This protocol is for a definitive randomised controlled trial of clinical and cost effectiveness comparing MBCT-SH with CBT-SH for people experiencing mild to moderate depression. Our primary hypothesis is that supported MBCT-SH, in comparison to supported CBT-SH, will lead to greater reductions in depressive symptom severity from baseline to post-intervention.

Secondary hypotheses are:

- MBCT-SH in comparison to CBT-SH will lead to greater reduction in depressive symptom severity from baseline to 6-month follow-up.
- A greater proportion of MBCT-SH participants will be in the non-clinical range for depressive symptoms than CBT-SH participants at post-intervention (i.e. remission) and 6-month follow-up (i.e. recovery).
- MBCT-SH in comparison to CBT-SH will lead to greater improvements in mindfulness, generalised anxiety, work and social adjustment and well-being from baseline to post-intervention and from baseline to 6-month follow-up.
- Treatment completion rates will be higher for MBCT-SH than CBT-SH.
- Depressive symptom severity outcomes will be mediated by treatment completion.
- MBCT-SH will be cost-effective in comparison to CBT-SH at follow-up.
- MBCT-SH will be a safe alternative to CBT-SH, with a similarly low incidence of serious adverse events during therapy and lasting negative effects of therapy.

In the event that the primary hypothesis of MBCT-SH superiority over CBT-SH on the primary outcome is not supported an additional exploratory analysis will be carried out to explore non-inferiority of MBCT-SH compared to CBT-SH.

A qualitative component will close the evidence gap concerning reasons for treatment non-completion in IAPT services by identifying facilitators and barriers to treatment completion in both arms. Qualitative interviews will be conducted using the Change Interview [19] to ascertain facilitators and barriers to treatment completion for each intervention. Questions were added to the end of interview following advice from the study’s lived experience public and patient involvement (PPI) consultation panel. These questions enquire about participants’ experiences of their allocated intervention beyond depressive symptom change.

## Methods/design

### Design and sample size

This is a parallel-group, superiority, pragmatic, randomised controlled trial with 1:1 allocation to MBCT-SH or CBT-SH, with blinded assessments at all time points. Participants will be blind to the hypothesised direction of effects. We will randomly allocate 410 people meeting eligibility criteria for major depressive disorder or mixed anxiety and depression to receive MBCT-SH or CBT-SH, along with six sessions of support from a PWP. Participants will complete measures at baseline (time 0), 16 weeks post-randomisation (post-intervention, time 1) and 42 weeks post-randomisation (6-month follow-up, time 2). In addition, 24 participants will be interviewed about their experiences of both self-help interventions (12 participants per arm) at time 1 with a focus on better understanding barriers and facilitators to engaging in self-help interventions in IAPT services and investigating any negative experiences or effects of treatments.

The sample size was based on detecting a between-group effect size of 0.36 based on the difference between the reported between-group effect of CBT-SH (0.42) [20] and the reported between-group effect of MBCT-SH (0.78) [21]. Recruiting 205 patients into each arm would provide 90% power to detect a between-group difference of 0.36 with a 5% alpha and a two-sided *t* test whilst allowing for 20% attrition at post-intervention (as found in our pilot study). Therefore, a total sample size of 410 will be required.

The study design was informed through consultation with a PPI group chaired by a PPI co-applicant consultant (LL). Members of the PPI group had participated in a pilot study of the intervention or had lived experience of depression, mindfulness and/or CBT. The PPI group were instrumental in advising on a number of aspects of study design including the frequency and nature of support sessions and the study recruitment strategy and materials.

### Participants

Participants will be recruited through ten IAPT services across England. A list of sites can be requested through the corresponding author.

Inclusion criteria are that participants: 1) be aged 18 years or over; 2) meet diagnostic criteria on the revised Clinical Interview Schedule (CIS-R) [22] for a primary diagnosis of a depressive episode, mixed anxiety and depression, or non-specified mild neurotic disorder; 3) score 10 or more on the Patient Health Questionnaire 9 (PHQ-9) [23] at their initial IAPT assessment (cut-off indicating a probable major depressive episode); and 4) have sufficient literacy skills to read and understand the self-help materials.

Exclusion criteria are that participants: 1) have severe symptoms of depression (a score 20 or more on the PHQ-9); 2) a score of 4 on the CIS-R suicidality scale; and 3) express a strong preference (5/5) for one intervention over the other on the treatment preference question such that if randomised to the non-preferred intervention they would be likely to drop out of the intervention.

### Measures

#### Demographics

A questionnaire will be completed to collect demographic information.

#### Diagnostic status

The CIS-R [22] will be conducted at baseline (time 0) to ascertain diagnostic status. The CIS-R is routinely used in primary care mental health and IAPT research [1] and has been validated for telephone completion [24].

#### Primary outcome measure

Depression symptom severity will be measured using the PHQ-9 [23], a nine-item self-report measure of depression symptom severity used in all IAPT services with good sensitivity and specificity. Items are rated on a four-point scale. Scores under 10 are considered sub-clinical, 10–14 mild, 15–19 moderate and 20+ severe. The PHQ-9 will be administered at time 0, time 1 and time 2.

#### Secondary outcome measures

Generalised anxiety will be measured with the Generalised Anxiety Disorder 7 [25], a seven-item measure of generalised anxiety used in IAPT. Items are rated on a four-point scale and the measure has excellent psychometric properties [25]. This will be administered at time 0, time 1 and time 2.

Well-being will be measured with the short version of the Warwick Edinburgh Mental Well-being Scale [26], which consists of seven questions rated on a five-point scale designed to measure well-being. The scale has good psychometric properties and is used widely [27]. This measure was added following advice from the PPI consultation panel. This will be administered at time 0, time 1 and time 2.

Functioning will be measured with the Work and Social Adjustment Scale [28], a five-item measure of daily occupational and social functioning that is used routinely in IAPT. This will be administered at time 0, time 1 and time 2.

Mindfulness will be measured using the 15-item version of the Five-Facet Mindfulness Questionnaire [29]. This has excellent psychometric properties and is sensitive to change following MBCT [29]. This will be administered at time 0, time 1 and time 2.

The Change Interview [19] will be used to guide the qualitative component of the study. This is a widely used, semi-structured interview designed to explore participants’ experiences of psychological interventions and has been successfully used in previous studies by members our team. Questions about participants’ experiences of the effects of their allocated intervention on personally relevant outcomes (i.e. not necessarily restricted to depressive symptoms) have been added at the end of the interview following advice from the study’s lived experience consultation panel. The Change Interview will be conducted by the PPI consultant co-applicant on the study (LL) with the expectation that this will facilitate participants to be open about their experiences of the study and interventions. This will be conducted at time 1.

#### Health economic measures

For service use, an adapted version of the Adult Service Use Schedule (AD-SUS) will be used to measure individual-level all-cause hospital and community-based health and social care resource use. The AD-SUS was developed in previous research for use with people with common mental disorders [30, 31] and has been adapted for the purposes of this study. The baseline (time 0) AD-SUS will cover the period 3 months prior to randomisation. At the time 1 and time 2 follow-up interviews, the AD-SUS will cover the period since the last interview.

For health-related quality of life we will use the five-level version of the EuroQol five dimension (EQ-5D-5L) [32] generic, preference-based measure of health-related quality of life covering mobility, self-care, usual activities, pain/discomfort and anxiety/depression. We will use the recently developed five level version to maximise sensitivity [33]. This measure will be administered at time 0, time 1 and time 2.

#### Intervention evaluation measures and tools

The intervention expectation form will be used to assess expectation of benefit and treatment credibility at time 0.

The lasting negative effects questionnaire will be used to ask participants about any lasting negative effects of their allocated intervention at time 2.

The PWP rating scale will be used for participants to rate the quality/helpfulness of the sessions between the participant and their PWP at time 1.

Weekly diaries will record the extent to which participants are engaging with the self-help course each week during intervention delivery.

The engagement questionnaire (at the end of treatment) will record the extent to which participants engaged with the self-help course during the entire course of the intervention and will be administered at time 1.

The engagement questionnaire (at follow-up) will record the extent to which participants continued to engage with the self-help course following the end of the intervention and will be administered at time 2.

Session attendance will be the number of PWP sessions attended (0–6) and the duration of each support session.

Treatment completion is defined as attending at least 50% of the PWP sessions (i.e. attending at least three sessions).

### Procedure

**Fig. 1:**
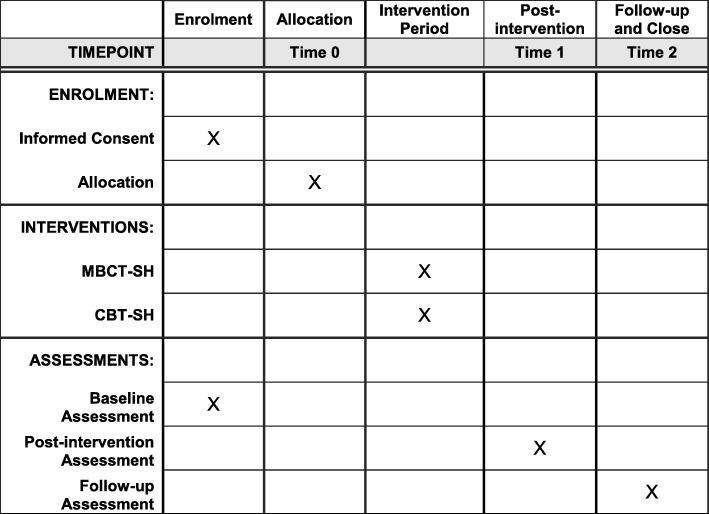
Schedule of enrolment, interventions, and assessments. CBT-SH cognitive behavioural therapy self-help, MBCT-SH mindfulness-based cognitive therapy self-help

See Fig. 1 for the SPIRIT schedule of enrolment, interventions and assessments. Patients with a diagnosis of depression will be sought through IAPT services. The study will also be advertised in general practitioner surgeries and elsewhere. Self-referrers will be guided to the study via their local IAPT service. Informed consent will be obtained from each participant. Potential participants will be given a copy of the study participant information sheet and will have the opportunity to discuss the study in person with the research assistant (RA) before signing the consent form (a copy of which can be obtained from the corresponding author).

Once the participant has consented to participate in the trial, the participant will complete the full set of baseline measures with an RA present in person or by telephone. Measures will be completed online or on paper, depending on participant preference. Participants who do not meet eligibility criteria at the baseline assessment will be referred back to the person who conducted their initial assessment for usual care to be offered by the service.

At the end of the baseline assessment, eligible participants will be randomised to either the MBCT-SH or CBT-SH arm. Participants will then be given their allocated self-help workbook. Randomisation will be stratified by centre and PHQ-9 score (mild or moderate) using random block length. Eligible participants will be randomly allocated using the Sealed Envelope [34] online service. The study statistician will use Sealed Envelope to set up and test the randomisation procedure, incorporating stratification by site and PHQ-9 severity category (mild or moderate) using random block length and 1:1 allocation. The statistician will not have any further involvement in the randomisation process. The RA will randomise participants by completing the online form with participant’s details. This will immediately show whether the participant is assigned to the MBCT-SH or CBT-SH arm and participants will be given their self-help workbook. Participants will not be told the hypotheses in relation to the arm to which they have been randomised.

Participants will then guide themselves through their allocated intervention over the 8-week course with six PWP support sessions. A maximum of 16 weeks is given for the intervention period to allow participants to complete their allocated 8-week course to take account of breaks for holidays, sickness and so forth. PWP support sessions may be offered by telephone or face-to-face, depending on usual practice in the service and participant preference. This mirrors the usual way in which self-help interventions are offered in IAPT (i.e. offering a self-help workbook alongside a limited number of PWP support sessions).

Participants will complete measures online (with a postal option) at 16 weeks post-randomisation (post-intervention, time 1) and 42 weeks post-randomisation (6-month follow-up, time 2). Participants will have the option to choose whether to complete the time 1 and time 2 measures with an RA present in person or by telephone (who will be blind to group allocation) or on their own. In the event that an RA is required to be present during time 1 or time 2 assessments and becomes unblinded the assessment will be completed by another, blinded RA. Where time 1 and time 2 assessments are not completed, participants will be contacted at weekly intervals for up to 1 month to remind them to complete these assessments, unless participants have discontinued from the study.

Twenty-four participants will be invited to take part in the qualitative Change Interview after their post-intervention (time 1) quantitative assessment is completed. Participants will be interviewed on a first-come, first-served basis, with 12 participants interviewed in each of two groups: 1) MBCT-SH intervention completers; and 2) CBT-SH intervention completers.

### Therapy protocols

#### MBCT-SH

The MBCT-SH workbook ‘The Mindful Way Workbook’ [35], written for clinical populations, presents MBCT as a self-help package. MBCT-SH participants will be given the workbook and will be asked to guide themselves through the self-help course within a 16-week time period (the time period determined in our pilot). As is routine at step 2, participants will be offered six PWP sessions to answer questions and provide encouragement.

PWPs currently train in CBT-SH. To match training between arms we will offer PWPs training in MBCT-SH. The training involves PWPs: 1) attending an MBCT group as a participant and guiding themselves through the MBCT-SH workbook, or completing the MBCT course using the workbook as a guide; and 2) attending a 2-day experiential mindfulness skills workshop. As is standard in delivering MBIs, PWPs will be encouraged to maintain their own personal mindfulness practice throughout the study, and this will be recorded using the PWP mindfulness practice record. Fortnightly MBCT-SH group supervision for PWPs will be provided.

#### CBT-SH

The CBT-SH workbook ‘Overcoming Low Mood and Depression’ [36] has evidence demonstrating its effectiveness in reducing depression symptom severity [37]. This workbook is often used at step 2 in IAPT.

Matched to the MBCT-SH condition, participants allocated to CBT-SH will be given a copy of their workbook and will be encouraged to guide themselves through the workbook within 16 weeks alongside six PWP sessions to answer questions and provide encouragement.

As in the MBCT-SH condition, fortnightly CBT-SH group supervision for PWPs will be provided.

#### Intervention fidelity and adherence

The same PWPs will deliver both interventions in order to minimise therapist effects as some PWPs achieve substantially better outcomes than others. In order to minimise therapeutic drift and therapy contamination the PWP protocols are detailed and there will be fortnightly supervision of PWPs. PWPs will be asked to audio record at least one complete MBCT-SH case and at least one complete CBT-SH case. A random 10% sample of recordings from each PWP will be rated for fidelity to the therapy protocols by a clinical psychologist trained in CBT and MBCT and who is independent of the PWP training and supervision.

Participants will be prompted each week by the RA to complete weekly diaries (online/paper versions) to record the amount of the workbook read and time spent engaged in intervention tasks.

### Serious adverse event monitoring

A protocol for identifying and independently assessing serious adverse events will ensure that such events are addressed in a timely fashion and responded to, including if a serious adverse event is classified as potentially study-related. Serious adverse events and their classification will be reported to the Data Safety Monitoring Board (DSMB) and Trial Steering Committee (TSC) and action will be taken as deemed necessary.

Serious and other adverse events will be discussed in intervention supervision (with CS or FJ) and in service-based clinical supervision in the relevant IAPT service. Where deemed in the best interests of participants, the study intervention may be discontinued and other treatment options may be recommended by the IAPT service.

## Planned data analysis

The primary analysis will be a quantitative analysis of the primary outcome at the primary end point (time 1) using an intention-to-treat (ITT) approach where participants are analysed as per their randomisation allocation regardless of treatment received.

Secondary analyses will consist of ITT analyses of the primary outcome at the follow-up time point (time 2) and all secondary outcomes. A per-protocol analysis will also be carried out for those participants receiving an adequate dose of their allocated intervention, defined as attending at least 50% of the PWP sessions (i.e. at least three sessions).

A descriptive summary of all measures will be provided by group (MBCT-SH and CBT-SH) and time point (time 0, time 1 and time 2) as appropriate. Comparisons between groups for all measures will be carried out using independent *t* tests and Chi-square testing for continuous and categorical data, respectively. Unstandardised effect sizes for the primary outcome and secondary outcomes will be estimated using linear mixed models with treatment group (MBCT-SH versus CBT-SH), time and a treatment group by time interaction entered as fixed factors; site, baseline PHQ-9 and baseline value of the outcome will be entered as covariates. Individual participants will be included in the analysis as random effects. Contrasts will be used as appropriate to estimate effects at different time points. A non-significant group by time interaction will imply common treatment effects at each time point.

Standardised (Cohen’s *d*) effect sizes for each outcome will be calculated by dividing the between-group unstandardized effect by the baseline pooled standard deviation. In addition, 95% confidence intervals will be calculated for all unstandardised estimates. Group differences in dichotomous outcomes at the different time points will be analysed in a similar way but using multilevel logistic regression models and baseline PHQ-9 scores. Baseline balance will be presented in the descriptive table broken down by study arm. No adjustment will be made for differences between covariates at baseline. Outliers will be removed if, and only if, they look erroneous. Scores at the extreme will not be removed if they are deemed to be true. The suitability of the assumption of approximate normality will be explored by plotting the residuals from this model. If normality is violated then transformations and non-parametric testing will be employed. A sensitivity analysis will be performed by carrying out the final analysis on the primary outcome, with and without any individual cases that were involved in violations of the protocol.

### Additional exploratory analysis

In the event that there is no evidence to support the primary hypothesis of MBCT-SH superiority over CBT-SH on our primary outcome, an additional exploratory analysis will be carried out to explore non-inferiority of MBCT-SH compared to CBT-SH. The analysis will be based on detecting a between-group non-inferiority margin of 2 points on the PHQ-9 with a one-sided α = 0.025. To operationalise this, a two-sided 95% confidence interval will be created around the effect size (MBCT-SH − CBT-SH) and we will conclude non-inferiority if the upper limit of the confidence interval is wholly below 2 PHQ-9 points for both the per-protocol and ITT analyses. The non-inferiority limit was set through consultation with service users and clinicians and looking at the literature [38, 39].

### Data entry accuracy and missing data

We aim to minimise missing data and data entry inaccuracies at the point of collection. The Qualtrics online survey software used to collect all data will automatically flag any unanswered questions, giving participants the chance to answer these. If a participant would prefer not to answer a question they can leave it unanswered for a second time and the software will proceed onto the next page. At the point of analysis, data will be summarised to look at patterns of missingness. Missing data will be replaced using multiple imputation as appropriate. Missing data will be assessed and if more than 5% of data is missing multiple imputation will be carried out followed by a sensitivity analysis. The sensitivity analysis will compare the results for a complete case analysis to the imputed data analysis. Multiple imputation will be carried out under the assumption the data is missing at random. Anonymised data will be stored on the Qualtrics platform and on password-protected NHS and university computers. Personal data will be stored securely in locked filing cabinets in NHS research offices and on password-protected NHS computers.

### Planned interim analysis and stopping rules

No interim analysis has been planned. The trial will be paused or stopped if deemed necessary by the DSMB.

### Multiple testing

There shall be no multiple testing.

### Economic evaluation

The economic evaluation will be conducted covering the period from time 0 to time 2 and will take the NHS/personal social services perspective preferred by NICE [40]. Data on resource use from the AD-SUS will be combined with unit costs to calculate the total costs of the MBCT-SH and CBT-SH groups. The cost of the two interventions, MBCT-SH and CBT-SH, will be calculated using a micro-costing approach [41] based on the salary of the PWPs, including relevant on-costs and overheads. Data on the number and duration of PWP contacts in the MBCT-SH and CBT-SH arms will be recorded using a proforma completed by PWPs. Data on indirect time, including preparation and supervision, will be collected directly from the PWPs. All other health and social care services will be costed using nationally applicable published unit costs, such as the NHS reference costs for hospital costs, the British National Formulary for medication and the Personal Social Services Research Unit costs of health and social care.

Costs and outcomes will be compared and presented in terms of mean differences and 95% confidence intervals obtained by non-parametric bootstrap regression to account for the non-normal distribution commonly found in economic data [42]. Cost effectiveness will be assessed through the calculation of incremental cost-effectiveness ratios and will be explored in terms of quality-adjusted life years calculated from the EQ-5D-5L and using the area under the curve approach [43]. Uncertainty will be explored using cost-effectiveness planes and cost-effectiveness acceptability curves based on the net-benefit approach [44, 45]. These curves are an alternative to confidence intervals around incremental cost-effectiveness ratios and show the probability that one intervention is cost effective compared to the other for a range of values that a decision maker would be willing to pay for an additional unit of an outcome. All economic analyses will include relevant baseline variables to provide a more relevant treatment-effect estimate [46]. The primary analysis will include those with complete data needed to be included in the economic evaluation. Sensitivity analyses will explore the impact of missing data.

### Qualitative data analysis

Qualitative data will be analysed using thematic analysis [47] to identify facilitators and barriers to treatment completion for each intervention arm. Analysis will be informed by the structure of the Change Interview [19], but it will also be able to identify inductively derived themes reflecting participants’ experiences.

## Discussion

CBT-SH, supported by a PWP, is the usual treatment offered in IAPT services to people with mild to moderate symptoms of depression. However, over half of people who complete this treatment remain depressed and, furthermore, less than half of IAPT service users complete treatment. This results in disappointing treatment outcomes for many service users and increasing costs for mental health services in primary care. Finding an alternative, more effective self-help treatment would lead to better outcomes for service users and a more efficient use of NHS resources.

MBCT-SH differs from CBT-SH in focus, approach and practice, but it may still be able to be delivered in IAPT services with support from PWPs. By teaching service users the ability to intentionally pay attention, non-judgementally, to current experience, MBIs aim to reduce rumination and worry, key components of depression, by teaching the application of mindfulness in everyday life and work. MBCT-SH, therefore, offers a potentially viable and more effective alternative to CBT-SH for improving both outcomes for service users and treatment completion rates.

This definitive randomised controlled trial directly compares MBCT-SH to CBT-SH with the primary aim of determining the clinical and cost effectiveness of MBCT-SH at reducing depression symptom severity compared to CBT-SH. There is also much less known about the effectiveness of supported self-help mindfulness-based interventions in comparison to their in-person group-based counterparts and so this trial will advance understanding in this area. If the primary superiority hypothesis is not supported, the exploratory non-inferiority analysis will help elucidate if MBCT-SH may be non-inferior to current best practice (i.e. CBT-SH) and therefore findings will be of clinical relevance. Findings from the Change Interview will highlight facilitators and barriers to engagement in both MBCT-SH and CBT-SH and will hopefully lead to recommendations for maximising engagement to both these interventions.

The results of this trial will provide valuable evidence to inform clinical practice and commissioners of services. This could enable a greater choice in effective treatment options for IAPT service users, help elucidate issues around why some people are unable to complete treatment and allow for a more efficient use of NHS resources. The results will also provide evidence more generally about mindfulness-based interventions, their clinical and cost effectiveness, and the mechanisms of change which underpin them.

## Trial status

At the time of manuscript submission, recruitment for this study was ongoing. Recruitment started on 6 November 2017 and is due to be completed on 31 January 2020. The first patient was randomised on 24 November 2017. This is protocol version 1 (18 January 2020). The trial sponsor is the Sussex Partnership NHS Foundation Trust (ResearchGovernance@sussexpartnership.nhs.uk).

## Supplementary information

**Additional file 1.** SPIRIT 2013 checklist: recommended items to address in a clinical trial protocol and related documents.

## Data Availability

The datasets created for the current study will be available from the corresponding author on reasonable request.

## Abbreviations

AD-SUS: Adult Service Use Schedule
CBT: Cognitive Behavioural Therapy
CBT-SH: Cognitive behavioural therapy self-help
CIS-R: Revised Clinical Interview Schedule
DSMB: Data Safety Monitoring Board
EQ-5D-5L: Five-level, five-dimension EuroQol measure
IAPT: Improving access to psychological therapies
ITT: Intention-to-treat
MBCT: Mindfulness-based cognitive therapy
MBCT-SH: Mindfulness-based cognitive therapy self-help
MBI: Mindfulness-based intervention
NHS: National Health Service
NICE: National Institute of Health and Care Excellence
PHQ-9: Patient Health Questionnaire 9
PPI: Public and patient involvement
PWP: Psychological well-being practitioner
RA: Research assistant
TSC: Trial Steering Committee
